# Supplementary figures and images for: Protein Arginine N-methyltransferases 5 and 7 Promote HIV-1 Production
Source: Viruses. 2020 Mar 23;12(3):355. doi: 10.3390/v12030355 (PMC7150949; doi:10.3390/v12030355)

Figure S1

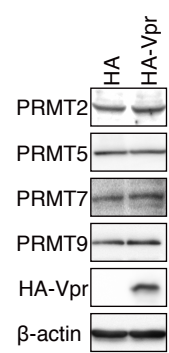

Figure S2

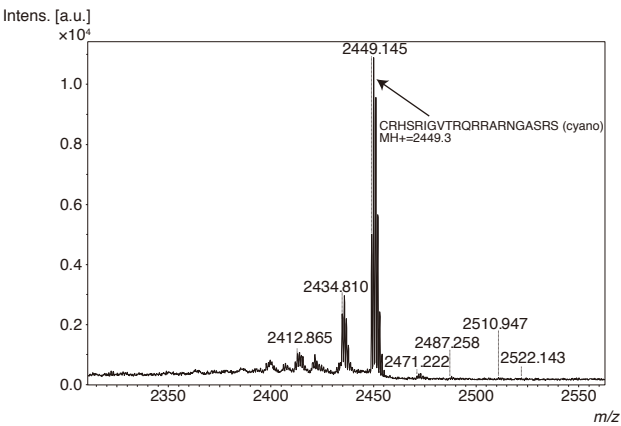

Figure S3

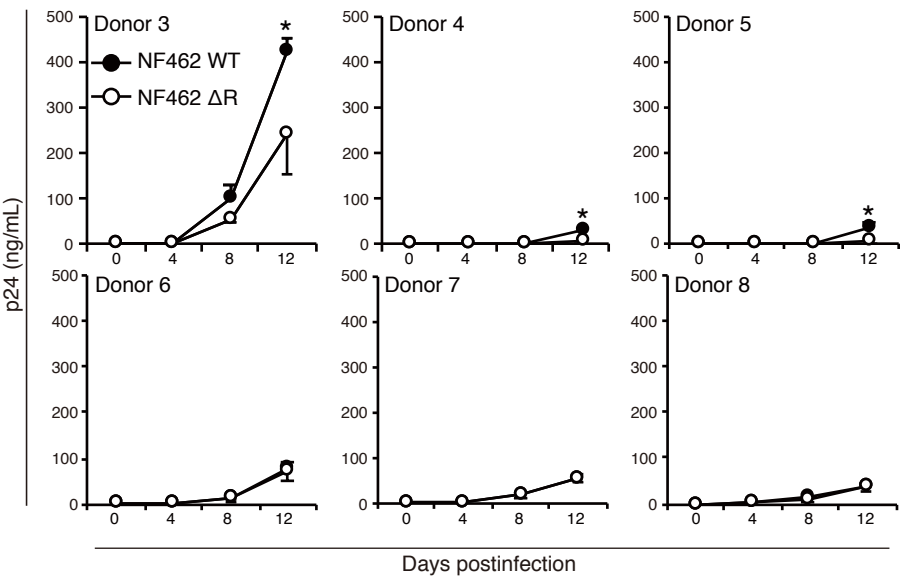

Supplement: Supplementary file 1 [file viruses-12-00355-s001.pdf]
